# Genomic and Metabolic Insights into Denitrification, Sulfur Oxidation, and Multidrug Efflux Pump Mechanisms in the Bacterium *Rhodoferax sediminis* sp. nov.

**DOI:** 10.3390/microorganisms8020262

**Published:** 2020-02-15

**Authors:** Chun-Zhi Jin, Ye Zhuo, Xuewen Wu, So-Ra Ko, Taihua Li, Feng-Jie Jin, Chi-Yong Ahn, Hee-Mock Oh, Hyung-Gwan Lee, Long Jin

**Affiliations:** 1 Co-Innovation Centre for Sustainable Forestry in Southern China, College of Biology and the Environment, Nanjing Forestry University, Nanjing 210-037, China; chunsik@kribb.re.kr (C.-Z.J.); taehwali@njfu.edu.cn (T.L.); jinfj@njfu.edu.cn (F.-J.J.); 2 Industrial Biomaterial Research Center, Korea Research Institute of Bioscience & Biotechnology (KRIBB), Daejeon 34141, Korea; 3 Cell Factory Research Centre, Korea Research Institute of Bioscience & Biotechnology (KRIBB), Daejeon 34141, Korea; poungcha@kribb.re.kr (S.-R.K.); cyahn@kribb.re.kr (C.-Y.A.); heemock@kribb.re.kr (H.-M.O.); trustin@kribb.re.kr (H.-G.L.)

**Keywords:** *Rhodoferax*, *Rhodoferax sediminis*, denitrification, sulfur oxidation, RND efflux systems

## Abstract

This genus contains both phototrophs and nonphototrophic members. Here, we present a high-quality complete genome of the strain CHu59-6-5^T^, isolated from a freshwater sediment. The circular chromosome (4.39 Mbp) of the strain CHu59-6-5^T^ has 64.4% G+C content and contains 4240 genes, of which a total of 3918 genes (92.4%) were functionally assigned to the COG (clusters of orthologous groups) database. Functional genes for denitrification (*narGHJI*, *nirK* and q*nor*) were identified on the genomes of the strain CHu59-6-5^T^, except for N_2_O reductase (*nos*) genes for the final step of denitrification. Genes (*soxBXAZY*) for encoding sulfur oxidation proteins were identified, and the *FSD* and *soxF* genes encoding the monomeric flavoproteins which have sulfide dehydrogenase activities were also detected. Lastly, genes for the assembly of two different RND (resistance-nodulation division) type efflux systems and one ABC (ATP-binding cassette) type efflux system were identified in the *Rhodoferax sediminis* CHu59-6-5^T^. Phylogenetic analysis based on 16S rRNA sequences and Average Nucleotide Identities (ANI) support the idea that the strain CHu59-6-5^T^ has a close relationship to the genus *Rhodoferax.* A polyphasic study was done to establish the taxonomic status of the strain CHu59-6-5^T^. Based on these data, we proposed that the isolate be classified to the genus *Rhodoferax* as *Rhodoferax sediminis* sp. nov. with isolate CHu59-6-5^T^.

## 1. Introduction

*Rhodoferax* species are frequently found in stagnant aquatic environments exposed to light [1], and some have been isolated from fresh water, sewage, sludge and sediments [2,3,4,5,6,7]. In the case of *Rhodoferax antarcticus*, strains were first isolated from microbial mats collected from saline ponds [2]. *Rhodoferax* growth occurs in a temperature range from 2 to 35 °C [1,2,3,4,5,6,7]. *Rhodoferax fermentans*, *Rhodoferax ferrireducens*, *Rhodoferax koreense*, *Rhodoferax lacus* and *Rhodoferax bucti* are mesophilic species with an optimal growth temperature between 25 and 30 °C [1,3,5,6,7]. The other two species, *R. antarcticus* and *Rhodoferax saidenbachensis*, are psychrotolerant with optimal growth temperatures ranging from 15 to 20 °C, but they are capable of growth at temperatures near 0 °C [2,4]. *Rhodoferax* is also well known for its diverse metabolic pathways. Members of the *Rhodoferax* species are able to grow phototrophically, aerobically and anaerobically. Two species in this genus, *R. fermentans* and *R. antarcticus*, grow photoheterotrophically using carbon sources such as acetate, pyruvate, lactate and succinate. Genomic features that provide photosynthesis gene clusters of the *R. antarcticus* have already been reported [8]. In contrast, *R. ferrireducens* is able to grow anaerobically using organic electron donors to reduce ferric iron (Fe^+3^) to ferrous (Fe^+2^) [1,2,3,4,5,6,7]. As of 2019, seven genomes have been sequenced from the genus *Rhodoferax*. Some of them contain the genes for the RuBisCo (ribulose 1, 5-bisphosphate carboxylase/oxygenase), several heavy metal resisting genes, and putative arsenite efflux pump genes [9]. Recently, the species *R. ferrireducens* was applied to the area of sustainable energy microbial fuel cells (MFC), where a bacterial suspension was used as a source of electrons for the bacteria [10]. Additionally, *Rhodoferax* is one of most abundant groups in the lakes of northeastern Germany [11], understanding the ecology of the genus *Rhodoferax* and their roles in geochemical cycles, as well as their physiology, would contribute to the understanding of its functions in environments. In the present study, a non-phototrophic member in this genus *Rhodoferax* obtained from freshwater was investigated at the genome and metabolic level, and a polyphasic approach was applied to establish its taxonomic status.

## 2. Material and Methods

### 2.1. Sampling, Isolation and Cultivation of the Strain

**Figure 1:**
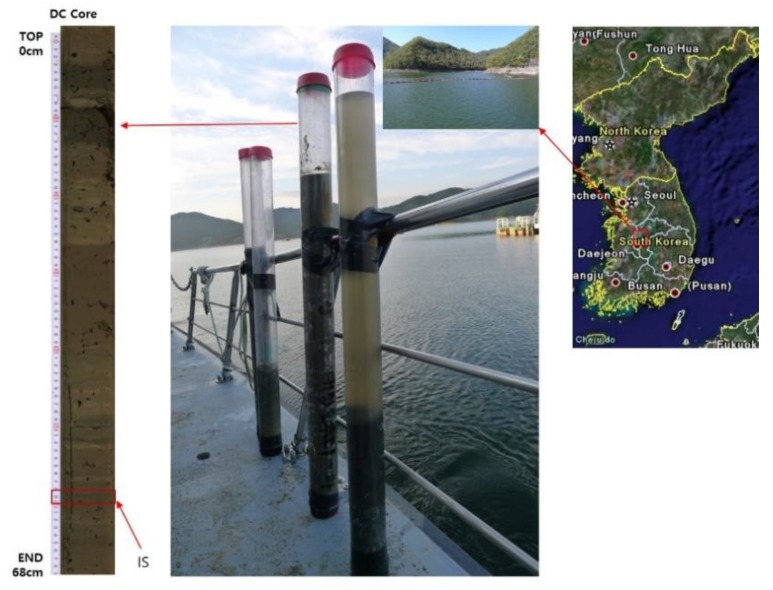
Sampling site and location of the Daechung Reservoir. Map shows the location of the sampling site on the shore in the vicinity of the Daechung Reservoir dam in the central region of South Korea. A 67 cm-long sediment core was collected at a 17 m water depth. IS, isolation section.

Strain CHu59-6-5^T^ was recovered from a 67 cm-long sediment core (36°22’30” N, 127°33’58” E) collected at a water depth of 17 m from the Daechung Reservoir using a modified gravity corer (Wildco, FL, USA) (Figure 1). The Daechung Reservoir is located in the central region of South Korea, which is a large branch-type lake, and the reservoir has a 72 m-high dam with a gross storage capacity of 1490 Mm^3^, where cyanobacterial blooms occur every year because of eutrophication and global warming [12,13,14,15]. Approximately one gram of sediment sample was applied to the serial dilution method in a 0.85% saline solution. A total of 100 µl aliquot of each serial dilution (10^−6^ or 10^−7^) was spread on onto modified 1/10 R2A medium (Difco, NJ, USA), which is specifically used for isolating aquatic bacteria [16] and incubated at 25 °C under aerobic heterotrophic conditions. A colorless colony CHu59-6-5^T^ was isolated after 6 days and routinely subcultured on an R2A agar at 30 °C for 48 h. For long term preservation of the culture, glycerol stocks (20% *v*/*v*) were prepared and stored at −70 °C. Strains were cultured on R2A agar for most physiological experiments.

### 2.2. Morphological, Physiological and Chemotaxonomic Characteristics 

The cell morphology was examined using transmission electron microscopy (Philips CM-20) after negative staining with 1 % (*w*/*v*) phosphotungstic acid, and the motility was checked in a phase-contrast microscope (Nikon Optiphot, 1000 × magnification) after 2 days of incubation in R2A at 30 °C. Gram staining was performed by using a Gram stain kit (Becton Dickinson) according to the manufacturer’s instructions. The activity of oxidase was tested using the oxidase reagent (bioMérieux), and catalase activity was determined by observing the production of O_2_ bubbles after dropping 3% (*w/v*) H_2_O_2_ on a fresh culture grown for 48 h on R2A medium [17]. Cell growth was investigated in different bacteriological media: R2A agar, tryptic soy agar (TSA; Difco, NJ, USA), Luria-Bertani (LB; Difco, NJ, USA) and nutrient agar (NA; Difco, NJ, USA). The growth temperature range was checked at 4, 8, 15, 20, 30, 37 and 42 °C. The pH range for growth was determined by measuring the OD values of R2A broth cultures after 3 days. Then, pH was adjusted to 5–10 at intervals of 1 pH unit with appropriate biological buffers [18,19]. Tolerance of NaCl was checked on R2A agar with different NaCl concentrations (0%–5%, *w/v*). Duplicated antibiotic-susceptibility was conducted using filter-paper disks containing the following: amikacin (30 µg·mL^−1^), ampicillin/sulbactam (20 µg·mL^−1^, 1:1), chloramphenicol (30 µg·mL^−1^), erythromycin (30 µg·mL^−1^), gentamicin (30 µg·mL^−1^), kanamycin (30 µg·ml^−1^), lincomycin (15 µg·mL^−1^), nalidixic acid (30 µg·mL^−1^), rifampicin (30 µg·mL^−1^), spectinomycin (25 µg·mL^−1^), streptomycin (25 µg·mL^−1^), teicoplanin (30 µg·mL^−1^), tetracycline (30 µg·mL^−1^, and vancomycin (30 µg·mL^−1^). Susceptibility results were recorded as positive at zones with diameters greater than 10 mm after incubation at 30 °C for 2 days. Carbon source utilization, enzyme activities and additional physiological and biochemical characterization were performed using API 20NE, API ID 32GN and API ZYM kits (bioMérieux, l’Etoile, France) and the Biolog GN2 MicroPlate following the manufacturer’s instructions.

For the comparative whole-cell fatty acid profile, strains CHu59-6-5^T^, *R. saidenbachensis* DSM 22694^T^, *R. lacus* KACC 18983^T^, *R. bucti* KCTC 62564^T^ and *R. koreense* KCTC 52288^T^ were cultured on R2A agar for 3 days at 30 °C. Cell harvesting standardization was done following the method described by Jin et al. [20], and the extracted fatty acids were analyzed by gas chromatography (GC) (Hewlett Packard 6890, Kyoto, Japan) and identified in the TSBA 6 (Trypticase Soy Broth Agar) database using the Sherlock software 6.1. Respiratory isoprenoid quinones were extracted and analyzed by high performance liquid chromatography (HPLC) (Shimadzu, Kyoto, Japan) with the YMC-Pack ODS-A column, following the method described by Komagata and Suzuki [21]. Polar lipids were extracted according to the method of Tindall [22]. The biomass used for lipid extraction was obtained from cultures growing on R2A agar plates at 30 °C for 3 days. Several spraying reagents were applied for visualizing the spots on the two-dimensional thin layer chromatography (TLC) on silica gel 60 F254 plates (Merck): molybdatophosphoric acid for total spots, ninhydrin for aminolipids, molybdenum blue for phospholipids and alphanaphthol solution for glycolipids.

### 2.3. Genomic and Phylogenetic Analyses

Whole genomic DNA was extracted using a FastDNA^TM^ SPIN kit according to the manufacturer’s instructions. The 16S rRNA gene of strain Chu59-6-5^T^ was amplified by PCR (polymerase chain reaction) with the following universal primer sets: 27F (5’-AGA GTT TGA TCM TGG CTC AG-3’; *Escherichia coli* position 8-27) and 1492R (5’-TAC GGY TAC CTT GTT ACG ACT T-3’; *E. coli* position 1492-1510) [23], and identified in the server of EzBioCloud [24]. For the phylogenetic analysis, 16S rRNA gene sequences of strain CHu59-6-5^T^ and closely related species were aligned with clustal x  [25] and edited with bioedit [26]. Based on the aligned sequences, the phylogenetic tree was reconstructed using the following algorithms in the mega7 software [27]: neighbor-joining [28], maximum-parsimony [29] and maximum-likelihood methods [30]. In each case, bootstrap values were calculated based on 1000 resamplings of the sequences [31]. The whole genome sequencing was carried out using the SMRT (Single Molecule, Real-Time) platform at Novogene Biotechnology (Beijing, China) together with Illumina next-generation sequencing technology. The low-quality read filtration and assemblage of the draft genome were conducted using SMRT version 5.0.1. The genome annotation of strain CHu59-6-5^T^ was annotated in the RAST pipeline, and a sequence-based comparison was made using the SEED Viewer [32,33]. The predicted protein coding sequences (CDSs) were submitted to the COG (Clusters of Orthologous Groups) database (http://www.ncbi.nlm.nih.gov/COG/) to generate the functional category and summary statistics [34,35]. The average nucleotide identity (ANI) and digital DNA–DNA hybridization (dDDH) values were calculated using the OrthoANI tool in EZBioCloud web server [36]. 

## 3. Results and Discussion

### 3.1. Physiological Tests

Strain CHu59-6-5^T^ formed visible colonies at 48 h on R2A agar when incubated at 30 °C. Cell growth was found to occur at temperatures ranging from 4 to 37 °C, and quite good growth was observed at 4 °C after 10 days; however, no growth was observed at 0 or 42 °C. Growth was found to occur at pH 6–9; however, no growth was observed at pH 5 or 10. The cells were found to be Gram-stain-negative, catalase and oxidase-positive, motile by gliding, and short rod-shaped (Supplementary Figure S1). The cells were found to assimilate d-alanine, 2,3-butanediol, citrate, d-galactose (weakly), glycogen, *β*-hydroxybutyric acid, *α*-ketoglutaric acid, d,l-lactic acid, l-proline (weakly), succinamic acid, succinic acid monomethyl ester and turanose (weakly) but not the rest (API 20NE, API ID 32GN test strips and Biolog GN2). The cells were found to be positive for the following enzyme activities (API ZYM test strip): acid phosphatase (weakly), esterase (C4), esterase lipase (C8) and leucine arylamidase but not the rest (Table 1). The cells were found to be susceptible to amikacin (30 µg·mL^−1^), ampicillin/sulbactam (1:1; µg·mL^−1^), chloramphenicol (30 µg·mL^−1^), erythromycin (30 µg·mL^−1^), gentamicin (30 µg·mL^−1^), kanamycin (30 µg·mL^−1^), rifampicin (30 µg·mL^−1^), spectinomycin (25 µg·mL^−1^), streptomycin (25 µg·mL^−1^), teicoplanin (30 µg·mL^−1^), tetracycline (30 µg·mL^−1^) and but resistant to lincomycin (15 µg·mL^−1^), nalidixic acid (30 µg·mL^−1^) and vancomycin (30 µg·mL^−1^).

### 3.2. Chemotaxonomy

The major fatty acids (>10%) were sorted into three groups C_16:1_ *ω*7*c* and/or C_16:1_ *ω*6*c* (29.6%), C_16:0_ (21.6%) and C_17:0_ cyclo (15.0%) (Table 2). The profile of major fatty acids in the strain CHu59-6-5^T^ was consistent with the components in species from the genus *Rhodoferax*, although some qualitative and quantitative differences were found. The strain CHu59-6-5^T^ was observed to have quite large amounts of C_10:0_ 3-OH (6.4 %), C_17:0_ (6.9 %) and C_15:1_ *ω*6*c* (6.4 %), which were not found, or barely found, in the reference strains (Table 2). The major predominant respiratory ubiquinone was Q-8. The polar lipids consisted of phosphatidylethanolamine (PE), three unidentified phospholipids (PL1-3), two unidentified aminophospholipids (APL1-2) and four unidentified lipids (L1-4) (Supplementary Figure S2). The polar lipid profile of strain CHu59-6-5^T^ was similar to those of *R. saidenbachensis* DSM 22694^T^, *R. lacus* KACC 18983^T^, *R. bucti* KCTC 62564T and *R. koreense* KCTC 52288^T^ in that phosphatidylethanolamine and the unidentified polar lipid were the major polar lipids, but could be differentiated from those of the reference strains in the presence or absence of several other polar lipids.

### 3.3. Genomic Analysis: The Taxonomic Status

**Figure 2:**
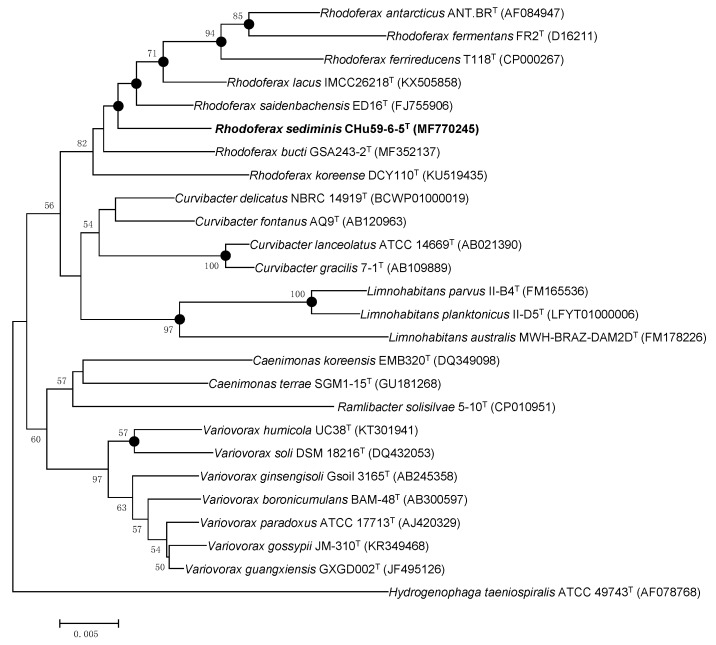
Phylogenetic tree constructed using the neighbor-joining method in mega 7 which depicts the phylogenetic relationship of strain CHu59-6-5^T^ among related taxa. The tree was reconstructed based on 1399 nucleotides. Numbers at branching points refer to bootstrap values (1000 resamplings, values above 50% shown). Bar, 0.5 substitution per 100 nt positions. Filled circles indicate that the corresponding nodes were also calculated in trees generated with the algorithms of maximum-likelihood and maximum-parsimony.

The 16S rRNA gene sequence (1478 nt) of strain CHu59-6-5^T^ was compared against the 16S rRNA gene sequences of representative species within the genus *Rhodoferax* and related genera. We used the EzTaxon-e server (www.ezbiocloud.net) to search for their closest members. The results show that strain CHu59-6-5^T^ shares a 98.8% pairwise similarity with *Rhodoferax saidenbachensis* ED16^T^, 98.4% with *Rhodoferax lacus* IMCC26218^T^, 98.2% with *Rhodoferax bucti* GSA243-2^T^ and less than 98.0% with other species in the genus *Rhodoferax*. The strain CHu59-6-5^T^ also shares high similarities with other species than just members of *Rhodoferax*: 98.1% with *Curvibacter delicates* NBRC 14919^T^ *Curvibacter fontanus* AQ9^T^, 97.8% with *Curvibacter fontanus* AQ9^T^, 97.5% with *Variovorax boronicumulans* BAM-48^T^ and 97.3% with *Variovorax paradoxus* NBRC 15149^T^. However, strain CHu59-6-5^T^ clustered clearly with the species of *Rhodoferax* from the topology of the phylogenetic tree (Figure 2). To determine genomic relatedness with other *Rhodoferax* species and its genomic characteristics, whole genome sequence of strain CHu59-6-5^T^ was obtained by the PacBio platform together with the Illumina MiSeq platform. The draft genome sequence of strain CHu59-6-5^T^ was deposited at DDBJ/EMBL/GenBank with the accession number CP036282. The genomic DNA G+C content of the strain was 64.4 mol%, which is within the range reported for the genus *Rhodoferax* (59.52–66.2 mol%). The observed ANI and dDDH values between strain CHu59-6-5^T^ and all species of *Rhodoferax* were 74.3%–76.9 % and 21.3%–23.3 % (Table 3), respectively, which were much lower than the species separation threshold of 95%–96% and in fact fall in the intergeneric range [37,38,39].

### 3.4. Genome Properties

**Figure 3:**
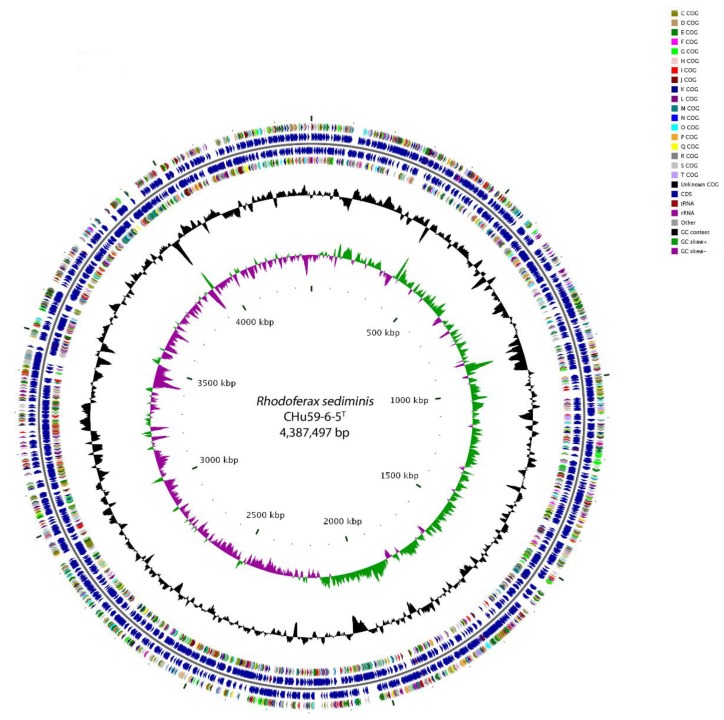
Graphic representation of circular genome plot of CHu59-6-5^T^. From the inner to the outer circle: The circles represent from outside to inside with the first and last circle showing the genomic position. The second and fifth circle shows the predicted protein coding sequences according to the COG (Clusters of Orthologous Group) database. The third and fourth circle shows protein-coding regions. The sixth circle represents variation in G + C content. The seventh circle shows a GC skew ([G − C]/[G + C]) plot of the genome.

The genome of strain CHu59-6-5^T^ consists of a single circular chromosome of 4,387,497 base pairs with a G+C content of 64.4% (Figure 3). Of the 4240 genes identified in the total genome, 4191 were protein-encoding genes, 49 were ribosomal or transfer RNAs, and 133 were putative pseudogenes (Table 3 and Supplementary Table S1). A total of 3918 genes (92.4%) were functionally assigned to the COG database. The distribution of the genes into the COG functional categories is presented in Table 3 and Supplementary Figure S3. Similar to the closely related *Rhodoferax* strains, abundant genes related to amino acid transport and metabolism (COG category E), transcription (COG category K), and energy production and conversion (COG category C) were observed. Unexpectedly, 27.7% of genes were assigned to the unknown function COG category in the genome of strain CHu59-6-5^T^ (Supplementary Table S2).

### 3.5. Carbon Metabolism

**Figure 4:**
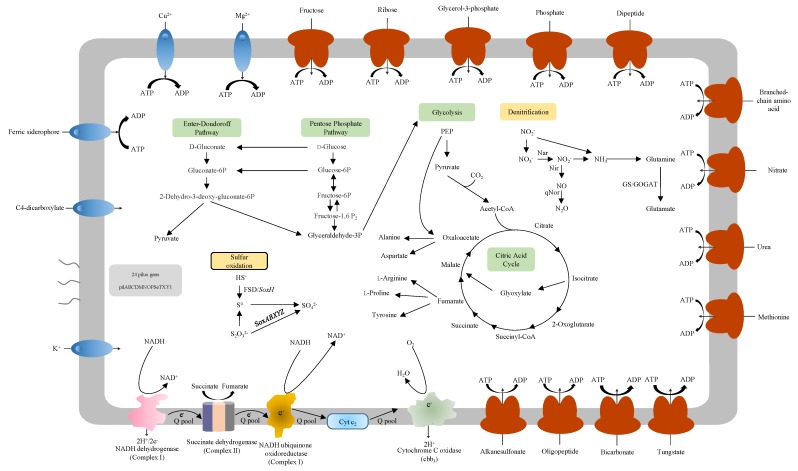
Model predictions for metabolism and some transporters of strain CHu59-6-5^T^. Nitrogen metabolic and sulfur oxidation pathways are indicated. Several transporters and electron transport systems are shown. Two electron chains, complex I (NADH dehydrogenase) and complex II (succinate dehydrogenase), were observed. Complex II usually parallel electron transport to complex I. A C-family heme-copper cbb3 oxidase, which presumably serves as the terminal electron acceptor during aerobic respiration, was identified. The genome also encodes genes related to the type IV pilus. PEP, phosphoenolpyruvate; GS/GOGAT: glutamine synthetase/glutamate oxoglutarate aminotransferase.

Two anoxygenic phototrophic species in the genus *Rhodoferax*, *R. fermentans* and *R. antarcticus* have been reported. Both members are confirmed to have gene clusters including genes encoding light-harvesting complex and RuBisCo (ribulose 1, 5-bisphosphate carboxylase/oxygenase) genes [1,2,8,40,41]. Genes of complete sets of glycolysis and the citric acid cycle were identified in the genome of strain CHu59-6-5^T^. Furthermore, genes for the Entner–Doudoroff pathway and pentose phosphate pathway were also detected (Figure 4). No genes for encoding RuBisCO (ribulose 1,5-bisphosphate carboxylase/oxygenase) or light-harvesting complex were found in the genome of strain CHu59-6-5^T^, which means that strain CHu59-6-5^T^ is not able to fix CO_2_ [41,42]. Strain CHu59-6-5^T^ contains genes encoding phosphoenolpyruvate (PEP) carboxylase (EC 4.1.1.31), but lacks genes encoding pyruvate–phosphate dikinase. This means that some other enzyme together with PEP carboxylase assists in adding CO_2_ to PEP [43].

### 3.6. Denitrification

Denitrification can take place in both terrestrial and marine ecosystems and is one of the main branches of the global nitrogen cycle supported by bacteria. Typically, denitrification occurs in anoxic environments, where the concentration of dissolved and available oxygen is depleted and nitrate (NO_3_^−^) or nitrite (NO_2_^−^) can be used as a substitute terminal electron acceptor instead oxygen [44,45,46].

**Figure 5:**
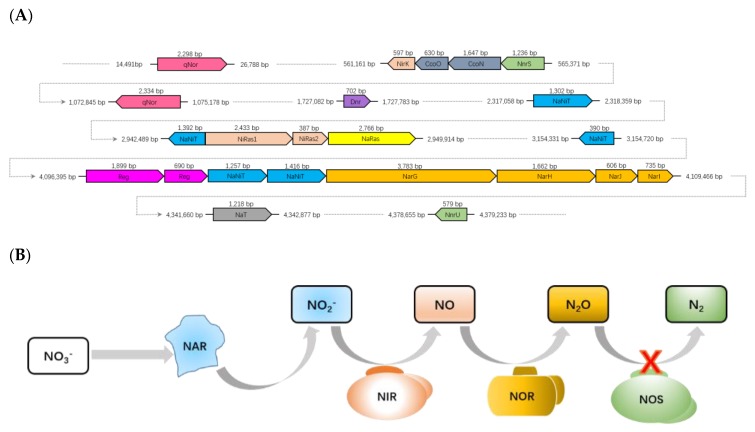
(**A**) Denitrification gene clusters located on draft genome of strain CHu59-6-5^T^. Color key: pink, *nor* genes; light orange, *nir* genes; blue-grey, *cco* genes (cytochrome *c* oxidase subunit); green, *nnr* genes (NO sensing protein (NnrS); denitrification regulatory protein (NnrU); white, *dnr* gene (NO responding transcriptional regulator); blue, *naNiT* genes (nitrate/nitrite transporter); yellow, *naRas* gene (nitrite reductase [NAD(P)H] large subunit (NaRas1), Nitrite reductase [NAD(P)H] small subunit (NaRas2); purple, *reg* genes; gold, *nar* genes; grey, *naT* gene (nitrate ABC transporter). (**B**) The substance transformation in the denitrification process was identified from the genome.

Key functional genes (*narGHJI*, *nirK* and q*nor*) for denitrification were identified on the genomes of the strain CHu59-6-5^T^ except for *Nos* genes. Currently, denitrification starts with a membrane bound (*narGHI*) in the mesophilic models of bacteria [47,48]. Genes encoding nitrate reductase (*narGHJI*) and nitrate/nitrite transporters (*naNiT*) were identified (Figure 5A). Nitrite produced in the cytoplasm by NarGHJI will then be secreted to the periplasm through a membrane transporter (NaNiT) and reduced to nitric oxide (NO) by copper-containing nitrite reductase (NirK). Strain CHu59-6-5^T^ possesses two quinone-dependent nitric oxide reductases (qNor). As the NO is highly toxic, it needs to be reduced to N_2_O immediately by a membrane-bound nitric oxide reductase (Nor) (Figure 5B). Depending on the type of Nor enzyme used, electrons for this reaction can be transferred by a periplasmic cytochrome c (*cNor*) or by quinones (qNor) [47,49,50,51]. Strain CHu59-6-5^T^ does not contain N2O reductase (Nos) genes for the final step of denitrification. In many denitrifying microorganisms, N_2_O is finally reduced to N_2_ by a periplasmic reductase (NosZ), in which a cytochrome c or a type I copper protein has to act as an electron donor [52]. However, the cases in which organisms without *nos* genes still can produce N_2_ gas have been studied, supporting the idea that a different type of N_2_O reductase needs to be discovered [53,54].

### 3.7. Sulfur Oxidation

Most of the sulfur-oxidizing bacteria are autotrophic and use reduced sulfur as electron donors for carbon dioxide fixation [55]; chemolithotrophic microorganisms obtain energy by oxidizing inorganic compounds for their structural components to survive, grow and reproduce [56,57]. Some inorganic forms of reduced sulfur (H_2_S/HS^−^, S^0^) can be oxidized by chemolithotrophic sulfur-oxidizing bacteria coupled to the reduction of oxygen (O_2_) or nitrate (NO_3_^−^) [56,58].

**Figure 6:**
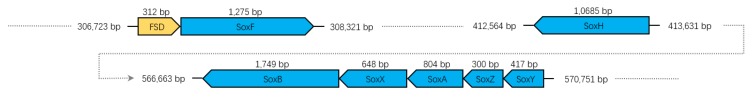
Sulfur oxidation gene cluster of strain CHu59-6-5^T^. Color key: golden, *FSD* gene (cytochrome *c* subunit of flavocytochrome *c* sulfide dehydrogenase); blue; *sox* genes.

The functional genes for sulfur oxidation (*sox*) participating in a thiosulfate-oxidizing multienzyme system include SoxXYZABDEFGH, which is able to oxidize various reduced sulfur compounds to sulfates [57]. The strain CHu59-6-5^T^ possesses genes (*soxBXAZY*) encoding for sulfur oxidation proteins (Figure 6), *soxB*, *soxXA* and *soxZY* genes are suggested to be essential to thiosulfate oxidation [59,60]. The SoxYZ complex appears as hetero- and homo-dimers with protein disulfide linked subunits, SoxB contains a dinuclear manganese cluster which is proposed to function as sulfate thiohydrolase and interacts with the SoxYZ complex [61,62]. The *FSD* and *soxF* gene encoding the monomeric flavoproteins which have sulfide dehydrogenase activities has also been detected. Those sulfur oxidation *Sox* systems indicate that the strain CHu59-6-5^T^ appears to be capable of thiosulfate oxidation to sulfate.

### 3.8. RND and ABC Efflux Systems

Bacterial efflux pumps are categorized into five families: ABC, the ATP-binding cassette superfamily; MFS, the major facilitator superfamily; MDR, the small multidrug resistance family; RND, the resistance-nodulation division superfamily; and MATE, the multidrug and toxic compound extrusion family [63,64]. RND efflux systems, a category of bacterial efflux pumps, play a prominent role in both intrinsic and acquired multidrug resistance identified in Gram-negative bacteria and catalyze the active efflux of a wide variety of antibacterial substrates including antibiotics and chemotherapeutic agents [65,66]. RND efflux systems are normally composed of three proteins: an inner membrane transporter, an outer membrane protein that functions as a pore, and a periplasmic protein that interacts with both the inner and outer membrane proteins to provide a conduit for the extrusion of small molecules [67].

**Figure 7:**
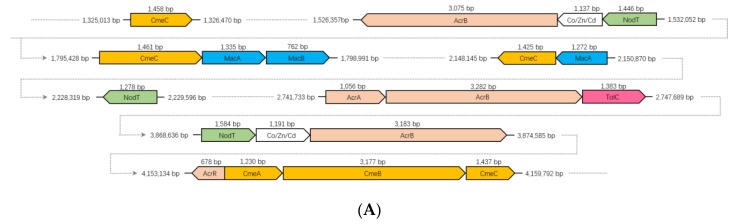

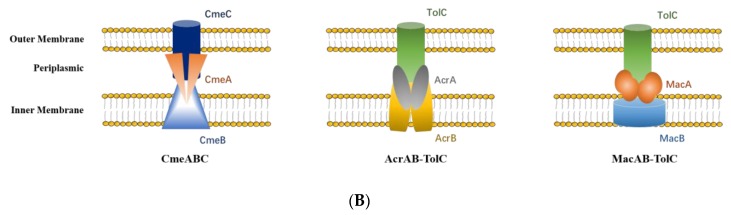
The gene clusters of RND and ABC type efflux pump in the genome of strain CHu59-6-5^T^ (**A**). Three types of tripartite efflux pump. From left to right, CmeABC pump, AcrAB-TolC pump and MacAB-TolC pump (**B**).

Two different RND type efflux systems and one ABC type efflux system have been detected in the strain CHu59-6-5^T^. The genome of the strain CHu59-6-5^T^ contains three-gene operon *cmeABC* (Figure 7A). *CmeABC* is also a tripartite efflux system composed of three proteins *CmeA*, *CmeB* and *CmeC*: the inner membrane protein *CmeB*, the periplasmic fusion protein *CmeA*, and the outer membrane protein *CmeC* [68]. A TetR family regulator gene *AcrR* was present just upstream of the operon. The *AcrR* regulator is known to act to repress the *AcrAB* operon in *E. coli* [69]. Except for the complete *CmeABC* gene cluster, three more *CmeC* genes encoding outer membrane lipoproteins were found throughout the genome. The genome also has been found to have complete genes for the AcrAB-TolC efflux pump, which has shown to be resistant to chloramphenicol, fluoroquinolone, tetracycline, novobiocin, rifampin, fusidic acid, nalidixic acid and *β*-lactam antibiotics [70]. Except for the complete AcrAB-TolC gene cluster, two more *AcrB* genes encoding the inner membrane proteins were detected through the genome. Interestingly, the genome of the strain CHu59-6-5^T^ possesses an ABC-type efflux pump MacAB-TolC (Figure 7B), which has resistance to a variety of macrolides, aminoglycosides and polymyxins [71]. TolC is an outer membrane protein which interacts with several inner membrane efflux pumps to expel antibiotics and export virulence factors from bacteria [72], and works in combination with other RND, ABC, and MFS efflux pumps [73,74]. The genome of strain CHu59-6-5^T^ possesses only one TolC gene, presumably working cooperatively as outer membrane proteins of AcrAB-TolC and MacAB-TolC. Those efflux systems may function complementarily with them and can work sequentially when one fails.

### 3.9. Motility

All the members of genus *Rhodoferax* are motile by polar flagella except for *Rhodoferax bucti* [1,2,3,4,5,6,7]. This genetic evidence of motility by flagella and type IV pili in *Rhodoferax antarcticus* was found in previous observations by Baker et al. [8], in which the genes coding proteins synthesizing polar flagella, fliEFGHIJKLMNPQR, were well studied.

The genome of strain CHu59-6-5^T^ contains genes for motility via IV pili but not flagella. Type IV pili, important virulence factors in many bacterial pathogens, generate motile forces called twitching or gliding motility in Bacteria and Archaea. The biogenesis of Type IV pili relies on macromolecular assemblies composed of 15 conserved proteins (PilC, PilD, PilE, PilF, PilG, PilH, PilI, PilJ, PilK, PilM, PilN, PilO, PilP, PilQ and PilW) in model Gram-negative bacteria [75,76]. The strain CHu59-6-5^T^ contains all 15 necessary genes for the proteins, and the type IV pilus genes in the strain CHu59-6-5^T^ are located in a more typical pattern throughout the chromosome (Supplementary Figure S4). Unfortunately, no pili of any type have been found in the cultured CHu59-6-5^T^ cells (Supplementary Figure S1), but the gliding motility was observed by a phase-contrast microscope.

## 4. Conclusions

To summarize our investigations, the strain CHu59-6-5^T^ from a freshwater sediment belonging to the genus *Rhodoferax* was studied through genome analysis, together with a polyphasic approach. The phylogenetic analysis indicated that the strain CHu59-6-5^T^ was affiliated closely with the species of *Rhodoferax*, based on the phylogenetic, genomic and physiological differences; thus, we propose the strain CHu59-6-5^T^ as a novel species, *Rhodoferax sediminis* sp. nov., in the family *Comamonadaceae*.

Unlike two phototrophs in the genus *Rhodoferax*, phototrophic systems were not detected. *Rhodoferax sediminis* CHu59-6-5^T^ possesses genes encoding for denitrification and nitrate ammonification related genes (GS/GOGAT pathway: glutamine synthetase/glutamate oxoglutarate aminotransferase) (data not shown). *Rhodoferax sediminis* CHu59-6-5^T^ also contains genes for sulfur oxidation and all the essential genes encoding for proteins of CmeABC, AcrAB-TolC and MacAB-TolC multidrug efflux systems. Besides this, the strain CHu59-6-5^T^ contains all necessary genes for type IV pilus proteins.

The genome sequence and comparative genome analyses of *Rhodoferax sediminis* CHu59-6-5^T^ provide a genetic blueprint and physiological characteristics which help us to understand the different metabolism and evolutionary life of the genus *Rhodoferax*.

Taxonomic Description of proposed *Rhodoferax sediminis* sp. nov.

*Etymology*: *Rhodoferax sediminis* (se.di′mi.nis. L. gen. n. *sediminis* of sediment).

Cells are Gram-stain-negative, motile by gliding and rods (0.8–1.3 µm long; 0.5–0.7 µm wide). Colonies grown on R2A agar are colorless. Cells are catalase and oxidase positive. Growth occurs on R2A at temperatures from 4 to 37 °C (optimum temperature 25–30 °C) but not 42 °C. The pH range for growth is from pH 6.0 to 9.0 (optimum pH 6-7) but not at pH 5.0 and 10.0. Growth occurs in the presence of 1% NaCl, but not with 2% or above. Cells are found to be positive for nitrate reduction but negative for indole production, glucose acidification, urease, aesculin hydrolysis, gelatin hydrolysis and β-galactosidase. Cells utilize d-alanine, 2,3-butanediol, citrate, d-galactose (weakly), d-glucose, β-hydroxybutyric acid, *α*-ketoglutaric acid, d,l-lactic acid, l-proline (weakly), succinamic acid, succinic acid monomethyl ester and turanose (weakly), but not acetic acid, *N*-acetyl-d-galactosamine, *N*-acetyl-glucosamine, *cis*-aconitic acid, adipate, adonitol, l-alaninamide, l-alanine, l-alanylglycine, l-asparagine, l-aspartic acid, γ-aminobutyric acid, 2-aminoethanol, l-arabinose, l-arabitol, bromosuccinic acid, caprate, dl-carnitine, d-cellobiose,*α*-cyclodextrin, dextrin, i-erythritol, formic acid, d-fructose, l-fructose, l-fucose, d-galactonic acid lactone, d-galacturonic acid, gentiobiose, d-gluconate, *α*-d-glucose-1-phosphate, d-glucose-6-phosphate, d-glucosaminic acid, α-d-glucose, glucuronamide, d-glucuronic acid, l-glutamic acid, glycerol, dl-α-glycerol phosphate, glycogen, glycyl l-aspartic acid, glycyl l-glutamic acid, l-histidine, hydroxy-l-proline, 3-hydroxybenzoate, 4-hydroxybenzoate, α-hydroxybutyric acid, γ-hydroxybutyric acid, *p*-hydroxyphenylacetic acid, inosine, inositol, *myo*-inositol, itaconate, α-ketobutyric acid, 2-ketogluconate, 5-ketogluconate, *α*-ketovaleric acid, *α*-d-lactose, lactulose, l-leucine, malate, malonate, maltose, d-mannitol, d-mannose, d-melibiose, methyl β-d-glucoside, l-ornithine, phenylacetate, l-phenylalanine, phenylethylamine, propionic acid, d-psicose, putrescine, l-pyroglutamic acid, pyruvic acid methyl ester, quinic acid, d-raffinose, l-rhamnose, d-ribose, d-saccharic acid, salicin, sebacic acid, d-serine, l-serine, d-sorbitol, suberate, succinic acid, sucrose, l-threonine, thymidine, d-trehalose, Tween 40, Tween 80, uridine, urocanic acid, valerate or xylitol. Cells are found to be positive for the following enzyme activities: acid phosphatase (weakly), esterase (C4), esterase lipase (C8) and leucine arylamidase. Cells are found to be negative for the following enzyme activities: *N*-acetyl-β-glucosaminidase, alkaline phosphatase, *α*-chymotrypsin, cystine arylamidase, α-fucosidase, α-galactosidase, β-galactosidase, α-glucosidase, β-glucosidase, β-glucuronidase, lipase (C14), *α*-mannosidase, naphthol-AS-BI-phosphohydrolase, trypsin and valine arylamidase. The only respiratory quinone is ubiquinone Q-8. The major polar lipids are phosphatidylethanolamine, two unidentified phospholipids and one unidentified aminophospholipid. The major fatty acids are grouped into three categories C_16:1_ *ω*7*c* and/or C_16:1_ *ω*6*c*, C_16:0_ and C_17:0_ cyclo. The G+C content of the DNA is 64.4 mol%. The strain type CHu59-6-5^T^ was isolated from a sediment sample of Dachung reservoir, Daejeon, Republic of Korea, and deposited at two different culture collections within the assigned numbers of KCTC 62554^T^ and JCM 32677^T^, respectively.

The GenBank/EMBL/DDBJ accession numbers for the 16S rRNA gene sequence the whole genome sequence of strain CHu59-6-5T are MF770245 and CP035503, respectively.

## Figures and Tables

**Table 1:** Features that differentiate strain CHu59-6-5^T^ from the most closely related species in the genus *Rhodoferax*.

Characteristic	CHu59-6-5^T^	*R. saidenbachensis* DSM 22694^T^	*R. lacus* KACC 18983^T^	*R. bucti* KCTC 62564^T^	*R. koreense* KCTC 52288^T^
Isolation source	Sediment	Sediment*	Freshwater^†^	Freshwater^ǂ^	Sludge^§^
Morphology	Short rods	Short rods*	Rods^†^	Curved rods^ǂ^	Rods^§^
Colony colour	Colourless	Colourless	Colourless	Peach brown	Colourless
Motility	+	+	+	-	+
Growth temperature	4–37	4–30*	4–30^†^	15–35^ǂ^	4–30^§^
Oxidase/catalase	+/+	+/−	+/+	−/+	−/+
Urease	−	+	+	−	+
Aesculin hydrolysis	−	−	+	+	−
Enzyme activity:					
alkaline phosphatase	−	+	+	+	+
esterase (C4)	+	+	+	+	−
esterase lipase (C8)	+	+	−	+	+
*α*-glucosidase	−	−	−	+	−
*α*-galactosidase	−	−	−	+	−
*β*-galactosidase	−	−	+	+	−
leucine arylamidase	+	−	+	+	+
naphthol-AS-BI-phosphohydrolase	−	+	−	+	+
Carbon utilization:					
l-alanine	+	−	−	−	+
l-arabinose	−	−	−	−	+
citrate	+	−	−	−	−
l-fucose	−	−	−	+	−
gluconate	−	−	−	−	+
d-glucose	+	+	+	−	+
histidine	−	−	−	−	+
3-hydroxy-benzoate	−	−	−	−	+
4-hydroxy-benzoate	−	−	−	−	+
3-hydroxy-butyrate	+	−	−	−	+
d,l-lactate	+	+	−	−	+
2-ketogluconate	−	−	−	+	+
5-ketogluconate	−	−	−	+	+
maltose	−	−	−	+	−
d-mannitol	−	+	−	+	+
d-mannose	−	+	−	−	-
d-melibiose	−	−	−	+	-
l-proline	−	−	−	−	+
d-ribose	−	−	−	+	+
d-sorbitol	−	−	−	+	−
d-sucrose	−	−	−	+	−
DNA G+C content (mol%)	64.4	60.3–61*	62.3^†^	61.2^ǂ^	60.3^§^

All data are from this study unless indicated. +, positive; −, negative. Note: * Data from Kaden et al. [4]; † Data from Park et al. [6]; ǂ Data from Zhou et al. [7]; § Data from Farh et al. [5].

**Table 2:** Cellular fatty acid compositions (%) of strain CHu59-6-5^T^ and the type strains of related species of the genus *Rhodoferax*.

Fatty Acids	CHu59-6-5^T^	*R. saidenbachensis* DSM 22694^T^	*R. lacus* KACC 18983^T^	*R. bucti* KCTC 62564^T^	*R. koreense* KCTC 52288^T^
**Saturated**					
C_11:0_	−	−	−	−	0.9
C_12:0_	3.0	1.3	1.1	0.5	12.0
C_13:0_	1.3	−	−	−	−
C_14:0_	2.9	0.2	0.7	0.3	1.7
C_15:0_	−	0.5	1.3	1.1	−
C_16:0_	21.6	33.6	28.2	29.3	24.7
C_17:0_	6.9	0.4	−	1.0	4.6
C_18:0_	−	1.2	1.3	2.0	0.3
**Unsaturated**					−
C_14:1_ *ω*5*c*	−	1.3	0.5	0.9	3.0
C_15:1_ *ω*6*c*	7.5	−	−	−	−
C_15:1_ *ω6c*	−	0.2	−	0.5	−
C_16:1_ *ω*5*c*	1.8	−	−	−	−
**Hydroxy**					−
C_8:0_ 3-OH	−	1.5	0.8	0.9	1.8
C_9:0_ 3-OH	0.9	−	−	−	−
C_10:0_ 3-OH	6.4	−	−	−	10.7
C_16:1_ 2-OH	1.2	−	−	−	−
C_17:0_ 3-OH	1.2	−	−	−	−
**Cyclo**					−
C_17:0_ cyclo	15.0	−	−	−	9.9
**Summed features^‡^**					−
3	29.6	53.6	59.3	60.2	26.5
8	0.8	6.1	4.2	2.8	2.3

Data are from the present study. −, not detected. Note: ‡ Summed Feature 3 contains C_16:1_ *ω*7*c* and/or C_16:1_ *ω*6*c*; summed feature 8 contains C_18:1_ *ω*7*c* and/or C_18:1_ *ω*6*c*.

**Table 3:** General features, and relationship of the genomes between strain CHu59-6-5^T^ and strains of its closely related species of the genus *Rhodoferax*.

Attribute	CHu59-6-5^T^	*R. saidenbachensis* DSM 22694^T^	*R. lacus* KACC 18983^T^	*R. bucti* KCTC 62564^T^	*R. koreense* KCTC 52288^T^	*R. ferrireducens* T118^T^	*R. fermentans* KACC 15304^T^
Genome size (bp)	4,387,497	4,264,855	4,900,405	3,673,501	5,895,641	4,969,784	4,467,741
G + C content (%)	64.4	60.9	62.3	61.2	66.2	59.9	56.9
N50	4,387,497	4,264,855	234,657	937,707	5,800,473	4,712,337	4,447,702
L50	1	1	6	2	1	1	1
Number of contigs	1	1	72	8	3	2	2
Number of coding sequences	4191	4030	4420	3436	5346	4339	4176
Number of tRNA	43	46	44	44	47	45	53
Number of rRNA	3	6	7	4	9	6	12
ANI (%)	100	75.4	74.9	74.4	76.9	75.2	74.3
dDDH (%)	100	22.1	21.3	23.3	20.9	22.3	22.4
GenBank Accession number	CP035503	CP019239	QFZK00000000	VAHD00000000	CP019236	CP000267	MTJN00000000

Data are from the present study unless indicated.

## Supplementary Materials

Supplementary materials can be found at https://www.mdpi.com/2076-2607/8/2/262/s1.
