# Supplementary material for: Genomic and Metabolic Insights into Denitrification, Sulfur Oxidation, and Multidrug Efflux Pump Mechanisms in the Bacterium Rhodoferax sediminis sp. nov
Source: Microorganisms. 2020 Feb 15;8(2):262. doi: 10.3390/microorganisms8020262 (PMC7074706; doi:10.3390/microorganisms8020262)

ARTICLE TITLE

Genomic and Metabolic Insights into Denitrification, Sulfur Oxidation, and Multidrug Efflux Pump Mechanisms in the Bacterium *Rhodoferax sediminis* sp. nov.

Chun-Zhi Jin[1, 2, ‡], Ye Zhuo [1, ‡], Xuewen Wu[1], So-Ra Ko[3], Taihua Li[1], Feng-Jie Jin[1], Chi-Yong Ahn[3], Hee-Mock Oh[3], Hyung-Gwan Lee[3], Long Jin[1, *]

[1] College of Biology and the Environment, Co-Innovation Centre for Sustainable Forestry in Southern China, Nanjing Forestry University, Nanjing 210-037, China

[2] Industrial Biomaterial Research Center, Korea Research Institute of Bioscience & Biotechnology (KRIBB), Daejeon 34141, Republic of Korea

[3] Cell Factory Research Centre, Korea Research Institute of Bioscience & Biotechnology (KRIBB), Daejeon 34141, Republic of Korea

Key words: *Rhodoferax*, *Rhodoferax sediminis*, denitrification, sulfur oxidation, RND efflux systems

[1] **Both authors contributed equally to this work.**

*** Corresponding author**
Long Jin
Tel: +86-25-8542-7210
Fax: +86-25-8542-7210
e-mail: isacckim@kaist.ac.kr

The GenBank/EMBL/DDBJ accession numbers for the 16S rRNA gene sequence the whole genome sequence of strain CHu59-6-5^T are MF770245 and CP035503, respectively.

Table S1. Features of the *Rhodoferax sediminis* CHu59-6-5^{T} genome

Attribute	Value	% of Total
Genome size (bp)	4,387,497	100
DNA coding (bp)	3,989,511	90.9
Total genes	4,240	100
CDSs	4,191	98.8
Protein-encoding genes	4,058	95.7
Pseudogenes (putative)	133	3.1
Genes assigned to COG	3,918	92.4
Genes with Pfam domains	3,740	88.2
Genes with transmembrane helices	946	22.3
Genes assigned to Mobilome	332	7.8
Gene island	12	0.3
CRISPR	3	0.1
RNA genes	49	1.2
tRNA genes	43	1
rRNA genes (5S/16S/23S)	3 (1/1/1)	0.1
Chromosome G + C content	64.35	-

Table S2. Distribution of genes in general COG categories of strain CHu59-6-5^{T} genome.

Functional code	Description	Genes	% of Genome content
A	RNA processing and modification	1	0.03
B	Chromatin structure and dynamics	4	0.10
C	Energy production and conversion	316	8.07
D	Cell cycle control, cell division, chromosome partitioning	23	0.59
E	Amino acid transport and metabolism	407	10.39
F	Nucleotide transport and metabolism	66	1.68
G	Carbohydrate transport and metabolism	168	4.29
H	Coenzyme transport and metabolism	131	3.34
I	Lipid transport and metabolism	192	4.90
J	Translation, ribosomal structure and biogenesis	162	4.13
K	Transcription	217	5.54
L	Replication, recombination and repair	199	5.08
M	Cell wall/membrane/envelope biogenesis	165	4.21
N	Cell motility	15	0.38
O	Posttranslational modification, protein turnover, chaperones	154	3.93
P	Inorganic ion transport and metabolism	217	5.54
Q	Secondary metabolites biosynthesis, transport and catabolism	114	2.91
S	Function unknown	1086	27.72
T	Signal transduction mechanisms	145	3.70
U	Intracellular trafficking, secretion, and vesicular transport	89	2.27
V	Defense mechanisms	47	1.20

Figure S1. Transmission electron micrograph of strain CHu59-6-5^T grown on R2A for 48 h. at 30 °C. Bar, 0.5 μm.

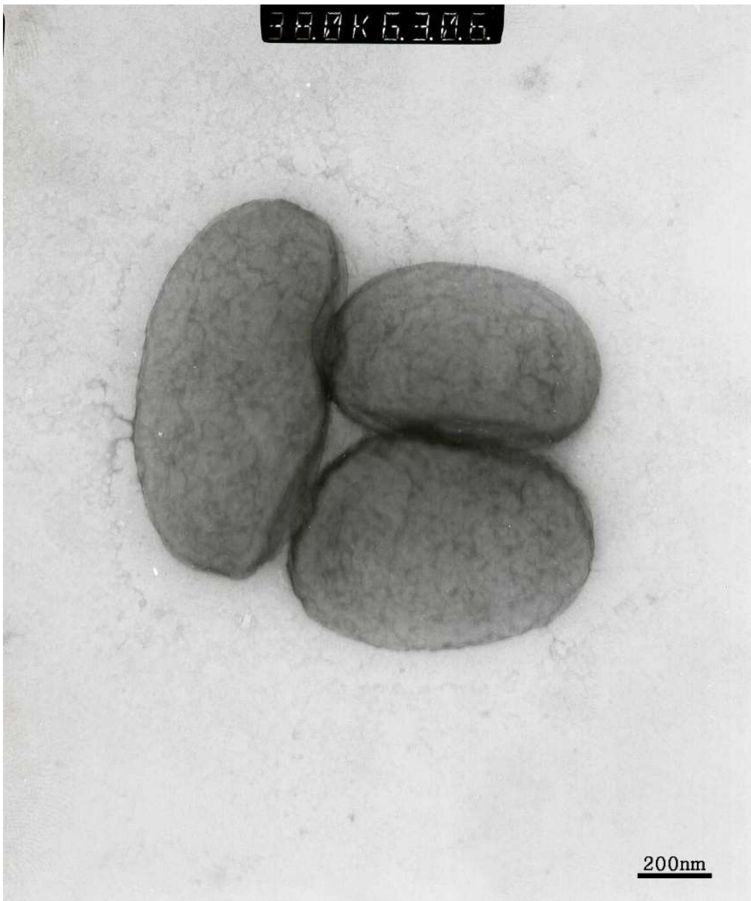

Figure S2. Polar lipid profile of strain CHu59-6-5^T. All the polar lipids were stained with molybdophosphoric acid (for total lipids), molybdenum blue (for phospholipids), and ninhydrin (for amino lipids). Solvents were as follows: first direction, chloroform/methanol/water (65:25:4, by vol.); second direction, chloroform/methanol/acetic acid/water (40:7.5:6:1.8, by vol.). PE, phosphatidylethnolamine; PL, unidentified phospholipids; APL, unidentified aminophospholipids L, unidentified lipids.

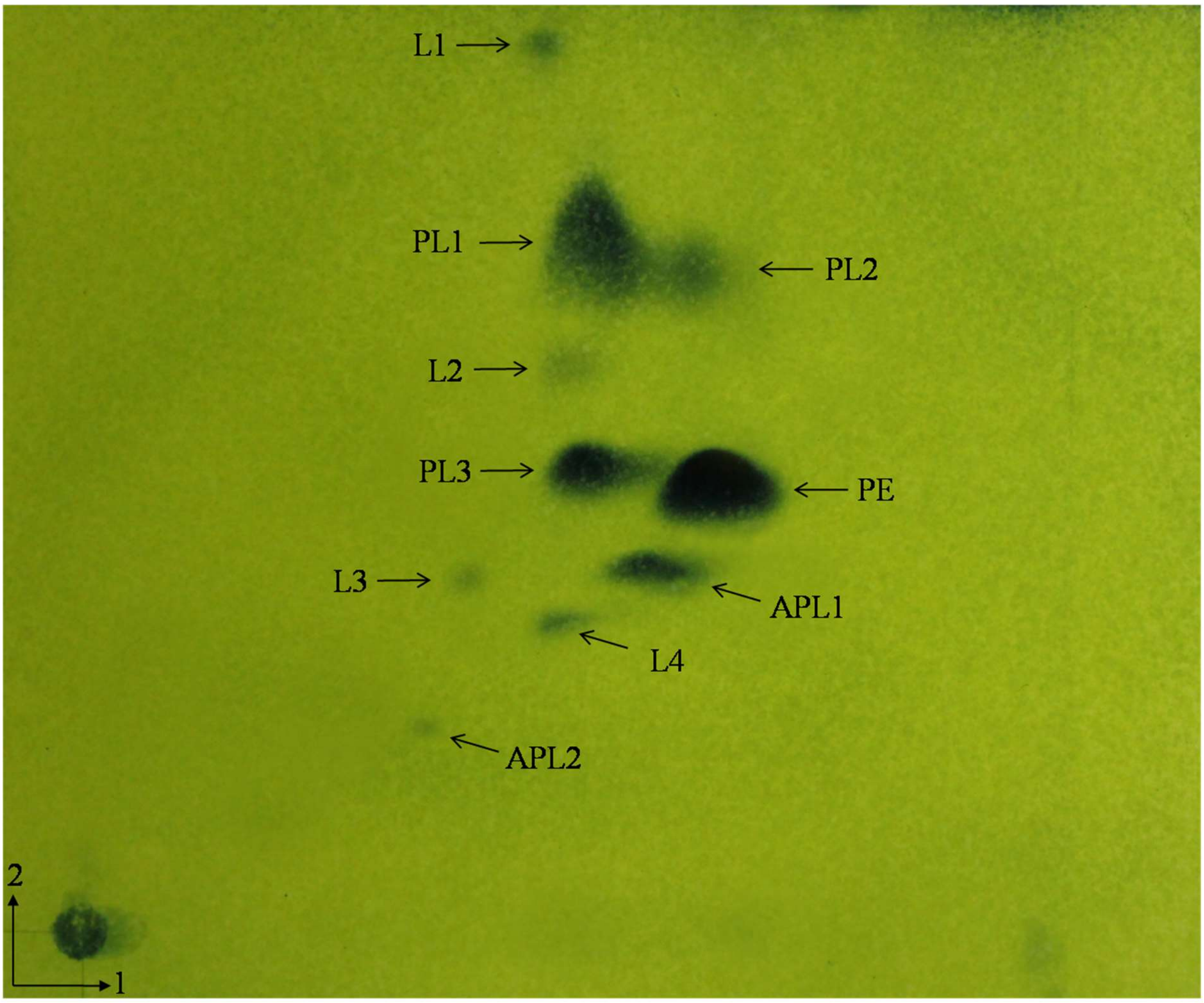

Figure S3. Distribution of predicted genes from whole genomes of strain CHu59-6-5^{T} and closely related species according to the COG functional categories.

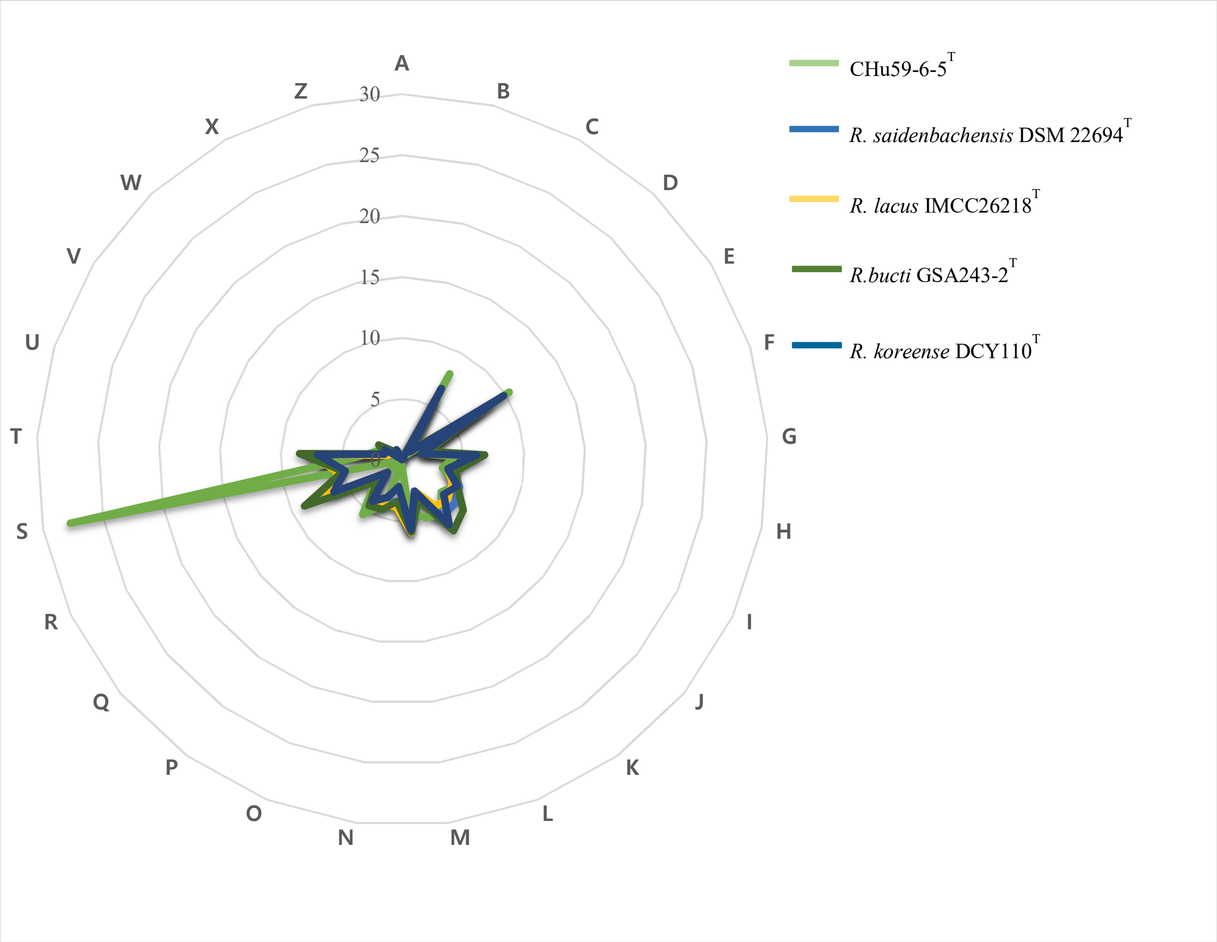

Figure S4. Linear representation of Type IV pilus genes for strain CHu59-6-5^{T}. Color key: purple, *pil* genes; blue, *fim* genes; orange, MTT genes.

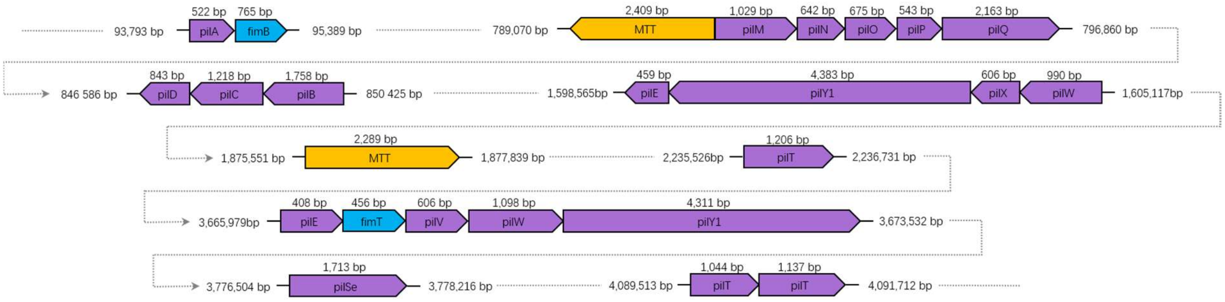

Supplement: Supplementary file 1 [file microorganisms-08-00262-s001.pdf]
